# Antenna Impedance Matching Using Deep Learning

**DOI:** 10.3390/s21206766

**Published:** 2021-10-12

**Authors:** Jae Hee Kim, Jinkyu Bang

**Affiliations:** 1 School of Electrical, Electronics and Communication Engineering, Korea University of Technology and Education, Cheonan 31253, Korea; jaehee@koreatech.ac.kr; 2 Department of Electrical and Electronic Engineering, Youngsan University, Yangsan 50510, Korea

**Keywords:** antenna impedance matching, artificial neural network, deep learning, input impedance (*S*_11_)

## Abstract

We propose a deep neural network (DNN) to determine the matching circuit parameters for antenna impedance matching. The DNN determines the element values of the matching circuit without requiring a mathematical description of matching methods, and it approximates feasible solutions even for unimplementable inputs. For matching, the magnitude and phase of impedance should be known in general. In contrast, the element values of the matching circuit can be determined only using the impedance magnitude using the proposed DNN. A gamma-matching circuit consisting of a series capacitor and a parallel capacitor was applied to a conventional inverted-F antenna for impedance matching. For learning, the magnitude of input impedance *S*_11_ of the antenna was extracted according to the element values of the matching circuit. A total of 377 training samples and 66 validation samples were obtained. The DNN was then constructed considering the magnitude of impedance *S*_11_ as the input and the element values of the matching circuit as the output. During training, the loss converged as the number of epochs increased. In addition, the desired matching values for unlearned square and triangular waves were obtained during testing.

## 1. Introduction

The most recent electronic devices support wireless communication, for which an antenna operating at a specific frequency band must be used. As the resonant frequency of an antenna is affected by its shape and surrounding materials in a device, the antenna must be modified whenever the device design is changed. To avoid antenna redesign, commercial communication devices can be used. However, such devices often include bulky external dipole antennas. In addition, the required operation frequency may not be available because commercial devices are intended for predefined frequencies, such as the ISM (industrial, scientific, and medical) band. If an antenna with a fixed shape could automatically operate at the desired frequency, the development time of wireless devices could be notably reduced along with the development cost of antennas.

Antennas used to tune the resonant frequency can be divided into reconfigurable and tunable antennas. Reconfigurable antennas [1,2,3,4,5] adjust the resonant frequency by changing their shape through a switch. Thus, small antennas or multiband antennas should often be reconfigurable given the difficulty to obtain a wide bandwidth. For instance, the tuning of the resonant frequency while reducing the size of a slot loop antenna has been achieved by using varactor diodes [3]. In addition, selection of the LTE (Long Term Evolution) band of 1.8 or 2.6 GHz has been achieved by inserting a PIN diode at the end of a loop antenna [4]. A reconfigurable antenna can change its radiation pattern by modifying its structure, providing high radiation efficiency depending on its shape. However, reconfigurable antennas are generally difficult to design given their complex structure.

On the other hand, tunable antennas operate at various frequency bands by changing the element values in the matching circuit [6,7,8,9,10,11,12]. As their structure is fixed and only the element values in the matching circuit change to obtain a resonant frequency, tunable antennas are simple to design. By applying a tunable matching circuit to an ultrawideband antenna, resonant frequencies from 1.8 to 2.8 GHz have been set at applied voltages from 0 to 23 V [9]. In addition, a tunable matching circuit has been applied to provide the required service frequency bandwidths for small antennas [12]. However, tunable antennas deliver suboptimal efficiency due to losses in the matching elements at any operating frequency [10]. To improve performance and properly tune the resonant frequency, the magnitude and phase of the antenna impedance should be accurately determined. In addition, the factor causing the change in the resonant frequency over the range of element values should be identified. Despite their simple structure, tunable antennas must be carefully designed by considering the antenna characteristics for the matching circuit.

Recently, machine learning has been applied to optimize antenna performance [13,14,15,16,17,18,19] and implement impedance matching [20,21,22]. A machine learning method can determine the element values without requiring a mathematical description of the matching circuit. In wireless power transmission, a neural network has recently been used to achieve the maximum efficiency [20,21]. Specifically, the matching element value according to the impedance of a wireless power transfer (WPT) coil was learned, and matching was performed automatically based on the measured impedance. The efficiency can be maximized by automatically compensating the matching value according to the distance between WPT coils. To date, however, no machine learning method has been devised for antenna impedance matching.

We propose a deep learning method that determines the element values of the matching circuit for a given magnitude of input impedance *S*_11_. The input is only the impedance magnitude, and the output is the corresponding element values of the matching circuit. Unlike the conventional approach, the proposed method determines the appropriate matching element values, and it can solve even unimplementable input impedances.

The remainder of this paper is organized as follows. Section 2 presents the antenna structure and matching circuit used in this study. Section 3 describes the method for acquiring input impedance *S*_11_ according to the capacitor values of the matching circuit. In Section 4, we introduce the proposed deep neural network (DNN) for antenna impedance matching. Section 5 reports the deep learning results and presents the corresponding discussion. Finally, we draw conclusions in Section 6.

## 2. Antenna and Matching Circuit

**Figure 1:**
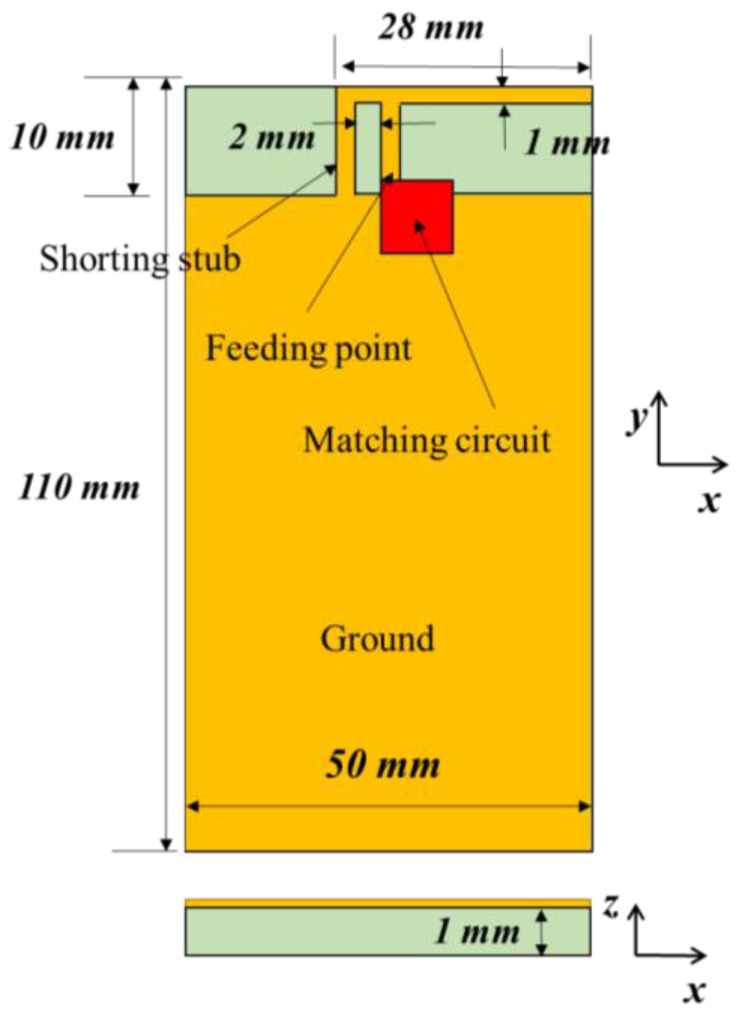
Antenna structure for impedance simulation.

Figure 1 shows the antenna structure to simulate the matching circuit effect. The basic structure is an inverted-F antenna, which is the most common type for mobile devices. The resonant frequency of the inverted-F antenna is determined by the length of the antenna, and the matching of the antenna is determined by the distance between the feeding point and the shorting stub. In general, the inverted F antenna is designed in the form of a meander line to include the length of the antenna in a narrow space in order to lower the resonant frequency. However, if there is not enough space for metal patterning, a matching circuit should be used to adjust the resonance frequency. The antenna is patterned on an FR4 substrate with a dielectric constant of 4.3 and a thickness of 1 mm. The obtained inverted-F antenna has a length of 28 mm and a height of 10 mm from the ground. The line width is 1 mm, and the shorting stub at the left end is connected to the ground. The feeding point is 2 mm from the shorting stub. The matching circuit is directly connected to the feeding point. The dimensions of the antenna are arbitrary. If there is no matching circuit, the antenna resonates at 1.9 GHz. The resonant frequency of the antenna can be tuned from 0.9 to 1.4 GHz using a matching circuit.

**Figure 2:**
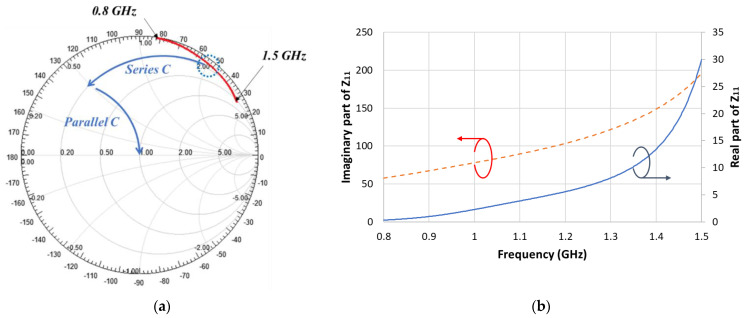
Simulated antenna impedance without matching circuit (**a**) Smith chart, (**b**) real and imaginary parts.

**Figure 3:**
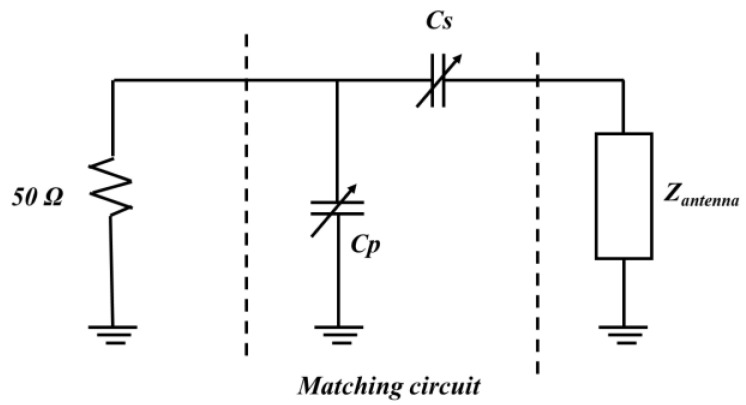
Gamma-matching circuit.

We performed simulations using Ansys HFSS (3D high-frequency simulation software). Figure 2 shows input impedance *S*_11_ of the antenna without a matching circuit on the Smith chart and the real part and imaginary part of the impedance for the frequency range of 0.8–1.5 GHz. As can be seen from Figure 2b, the imaginary part has values higher than 50 ohm as positive values. This means that in order to make resonance, the imaginary part should be compensationed through capacitors. The antenna impedances at the lowest and highest frequencies are located in the upper-right corner of the Smith chart. Therefore, the impedance can be matched by combining a series capacitor and a parallel capacitor, as shown in the gamma-matching circuit of Figure 3. Impedance matching is possible at the designed frequencies according to the capacitor values. Specifically, we used a series capacitor *C_S_* of 0.9–3.3 pF and parallel capacitor *C_P_* of 1–15 pF.

Matching should be applied for the capacitor values to match at the lowest and highest resonant frequencies of 0.9 and 1.4 GHz, respectively. For resonance at 1.4 GHz, the values of the series (*C_S_*) and parallel (*C_P_*) capacitors should be 0.9 and 3 pF, respectively. For resonance at 0.9 GHz, the respective values should be 3 and 20 pF. These values for the matching circuit were determined by mathematical calculations based on accurate information about the real and imaginary parts of the antenna impedance.

Although it is possible to measure *S*_11_ including its real and imaginary parts by using a network analyzer, expensive equipment is required. Instead, we propose a method for determining the matching element values using the *S*_11_ magnitude in a DNN. As the magnitude does not include phase information, an accurate matching value cannot be determined mathematically. However, through learning, the proposed method determines the matching element values solely from the input impedance magnitude.

## 3. Data Acquisition

**Figure 4:**
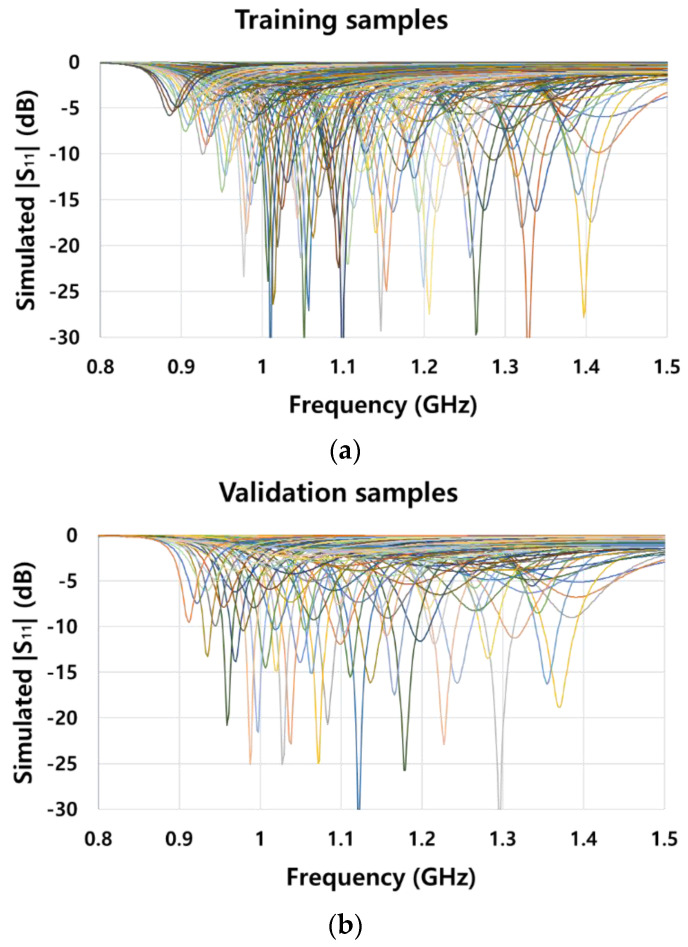
Magnitude of input impedance *S*_11_ for (**a**) all training and (**b**) all validation samples according to the combination of series capacitor *C_S_* and parallel capacitor *C_P_*.

The effectiveness of machine learning depends on the availability of large amounts of data. However, manually obtaining input impedance *S*_11_ according to the matching element values is time-consuming. Therefore, automated data acquisition should be performed. To this end, we linked MathWorks MATLAB and Ansys HFSS. In MATLAB, series capacitor *C_S_* and parallel capacitor *C_P_* were set as variables, and these values were linked with HFSS. According to the matching element values, the *S*_11_ magnitude was extracted as a text file. The matching element values for training are listed in Table 1, and those for validation are listed in Table 2. The magnitude of input impedance *S*_11_ is a scalar value ranging from 0 to 1 over 401 datapoints, corresponding to a frequency range from 0.8 to 1.5 GHz. For the training data, as 13 series capacitors and 29 parallel capacitors were used, 13 × 29 = 377 samples were obtained. In addition, the validation samples were 11 × 10 = 110. The postprocessing time to obtain *S*_11_ per setting of matching element values was 12 s, taking approximately 90 min to obtain all the training and validation samples. Figure 4 shows the *S*_11_ magnitude for all the training (Figure 4a) and validation (Figure 4b) samples. It is important to match the antenna impedance at the designed resonant frequency. The reason for graphing all samples in Figure 4 is to indicate that the resonant frequency of validation samples is different from the resonant frequency of the training samples. This is to investigate how well the DNN learns for these different resonant frequencies.

## 4. DNN Modeling and Training

**Figure 5:**
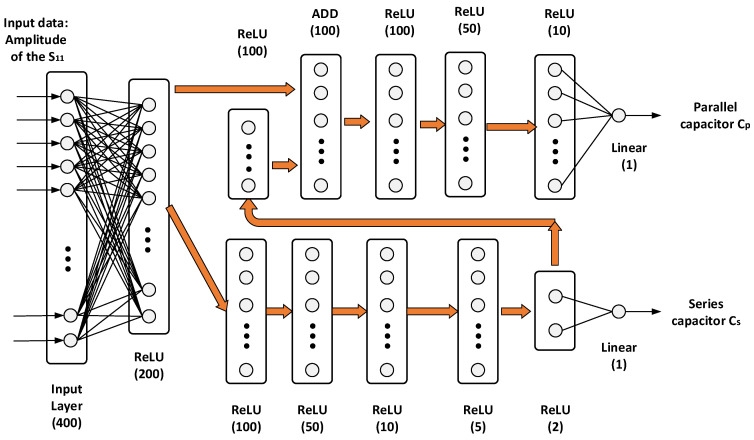
Architecture of the proposed DNN for antenna impedance matching.

Deep learning allows us to obtain the correct output for both learned data and previously unseen data. We used high-level Keras API in TensorFlow 2.0 to construct a DNN using Python. Figure 5 shows the structure of the proposed DNN. The input for deep learning is *S*_11_, whose magnitude is generally expressed in decibels. For implementation, the *S*_11_ magnitude was converted into a scalar value to normalize the input. In this study, the number of input samples was 401, with values ranging from 0 to 1 corresponding to frequencies from 0.8 to 1.5 GHz. The DNN output is given by the values of the series and parallel capacitors. As these values influence each other in the matching circuit, we considered two branches followed by addition (ADD layer) to reflect the influence, as shown in Figure 5. Each output value of the DNN for the corresponding capacitor value was obtained from one layer. As the DNN output should also be normalized, each capacitor value should be weighted. Input impedance *S*_11_ is highly sensitive to small values of the series capacitor. Therefore, we use the reciprocal of the series capacitor value as output. On the other hand, the value of the parallel capacitor was weighted by 0.1, as a larger value has a greater influence on the impedance. As a result, the weighted values of the two capacitors for training ranged from 0 to 1.5. The activation function of the output layer was linear, and the remaining layers used rectified linear unit (ReLU) activation to prevent the vanishing gradient problem. Each stage in the DNN implements a dense layer that fully connects the input and output neurons. As processing through the layers proceeded, the number of output neurons decreased. The number of neurons is expressed as a number in parentheses under the layer in Figure 5. The ADD layer functions to add two input values. RMSProp was used as the optimizer for learning DNN. The RMSProp does not simply accumulate gradients, but uses an exponentially weighted moving average to reflect the latest gradients larger. The loss function for DNN training was based on the mean squared error to perform optimization via root mean square propagation. The learning rate was set to 0.00005. For the DNN, 377 samples (Table 1) were used for training, and 110 samples (Table 2) were used for validation. Training proceeded for 2000 epochs with a batch size of 10.

**Figure 6:**
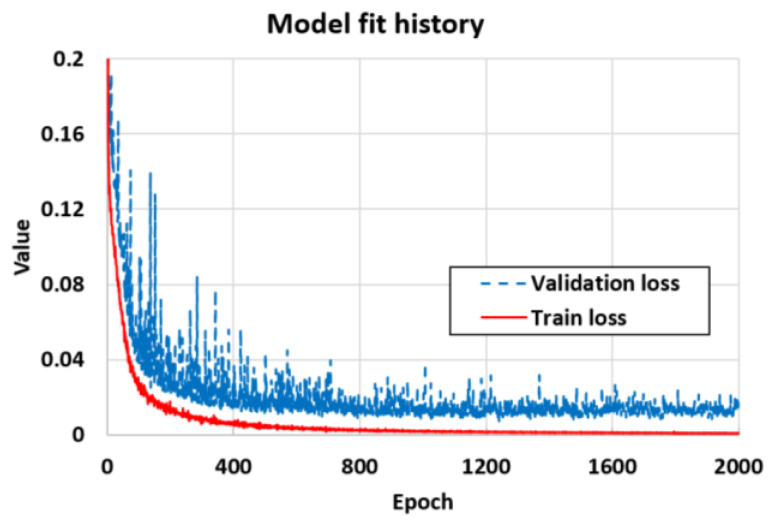
Training and validation losses according to epochs.

The loss throughout training is shown in Figure 6. As training proceeds, the loss values converged at 0.0010 for training and 0.013 for validation. In this study, it took approximately 10.5 min to train the DNN in a computer equipped with an Intel(R) Xeon(R) processor at 2.30 GHz and 16 GB memory.

## 5. Simulation Results and Discussion

**Figure 7:**
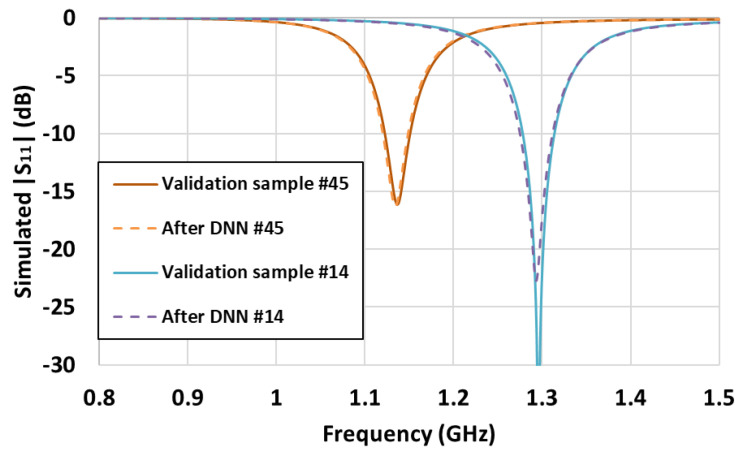
Comparison of results from ground truth and DNN output.

To validate the proposed DNN, two test sets with ground truths (i.e., calculated values) were considered. The selected capacitor values in the matching circuit are listed in Table 3. Samples with *S*_11_ magnitude up to 0.3 were selected, as antenna design requires *S*_11_ to be small. The *S*_11_ magnitudes from the test sets were used as input for the proposed DNN to obtain the corresponding capacitor values as outputs, as listed in Table 3. The capacitor values obtained from the DNN have some errors with respect to the calculated values. To analyze the effect of this error on antenna impedance matching, we conducted a simulation using the output capacitor values in HFSS. Figure 7 shows the comparison of *S*_11_ between the ground truths and DNN predictions, which are very similar.

**Figure 8:**
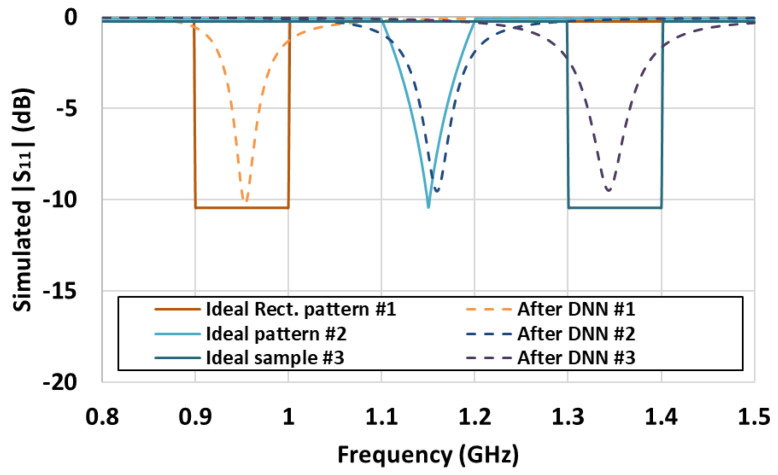
Comparison of ideal *S*_11_ waveform with that obtained from DNN results.

Using the trained DNN, the output was derived using ideal *S*_11_ patterns that cannot be implemented in practice as inputs. Table 4 lists the DNN outputs for three ideal patterns. The first ideal pattern is a square wave with *S*_11_ having magnitudes of 0.3 in 0.9–1.0 GHz and 0.97 in the other frequencies. The second ideal pattern is a triangular wave with the *S*_11_ magnitude decreasing linearly from 1.1 GHz until a minimum value of 0.3 at 1.15 GHz and then increasing linearly up to 1.2 GHz with a magnitude of 0.99. The third ideal pattern is also a square wave, but in a frequency range of 1.3–1.4 GHz. Figure 8 shows the comparison between the ideal *S*_11_ patterns and the simulated *S*_11_ patterns that use the output matching values obtained from the DNN. The DNN provides appropriate matching values for an ideal input. For the first, second, and third ideal patterns, the resonant frequencies were 0.95, 1.16, and 1.34 GHz, respectively. However, a completely consistent solution is infeasible because ideal patterns cannot be implemented in practice. Nevertheless, the DNN manages to determine the matching element values that approximate the desired *S*_11_ waveform.

Machine learning has been applied for impedance matching using neural networks, as listed in Table 5. However, those applications consider frequencies in the order of megahertz, which is relatively lower than the gigahertz band required for antenna impedance matching. Moreover, those applications are limited to implementable impedance patterns. On the other hand, the proposed DNN can perform antenna impedance matching in the gigahertz frequency band. Unlike conventional methods, it uses only the magnitude instead of the complex impedance value to learn antenna matching values. Moreover, reasonable capacitor values for antenna impedance matching can be obtained even for *S*_11_ magnitudes that cannot be implemented in practice. In reference papers [20,21], matching values for ideal inputs were not presented.

This study applied deep learning to antenna matching through simulation. For experimental verification, it is necessary to implement the tunable matching network with a control circuit including a DNN, and the magnitude of the *S*_11_ should be measured at the rear end of the matching circuit using a device that can measure the reflection coefficient. Using the switching value of tunable matching circuit and magnitude of the measured impedance, the applicability of the proposed DNN can be verified experimentally. It is also necessary to research whether the matching value is properly found when there is noise in the impedance data. The practical performance of the deep learning method in selecting the value of the matching circuit element is an interesting future work.

## 6. Conclusions

We proposed a DNN to determine the capacitor values in the circuit for antenna impedance matching. The matching circuit consists of a series capacitor and a parallel capacitor and is intended for an inverted-F antenna, which is often used in small wireless devices. *S*_11_ data were acquired by simulating the antenna structure for various capacitor values. Then, the DNN was constructed using the *S*_11_ magnitude as input and the capacitor values of the matching circuit as outputs. After training on 377 training samples and 64 validation samples, the DNN achieved a loss of 0.001. The trained DNN was then applied to *S*_11_ magnitudes of ideal square and triangular waves. The simulated *S*_11_ obtained from DNN outputs shows the desired resonant frequency even for physically impossible patterns, suggesting that deep learning can be used for robust antenna impedance matching.

## Figures and Tables

**Table 1:** Capacitor values in matching circuit for training.

Element	Values (pF)	No. Cases
Series capacitor *C_S_*	0.9, 1.1, 1.3, 1.5, 1.7, 1.9, 2.1, 2.3, 2.5, 2.7, 2.9, 3.1, 3.3	13
Parallel capacitor *C_P_*	1, 1.5, 2, 2.5, 3, 3.5, 4, 4.5, 5, 5.5, 6, 6.5, 7, 7.5, 8, 8.5, 9, 9.5, 10, 10.5, 11, 11.5, 12, 12.5, 13, 13.5, 14, 14.5, 15	29

**Table 2:** Capacitor values in matching circuit for validation.

Element	Values (pF)	No. Cases
Series capacitor *C_S_*	1, 1.2, 1.4, 1.6, 1.8, 2, 2.2, 2.4, 2.6, 2.8, 3	11
Parallel capacitor *C_P_*	1.1, 1.7, 2.3, 3.7, 5.5, 7.5, 9.5, 11.5, 13.5, 16	10

**Table 3:** Capacitor values in matching circuit for validation.

Ground Truth	DNN Output	Sample
*C_S_* = 1.2 pF	*C_S_* = 1.2191441 pF	14
*C_P_* = 5.5 pF	*C_P_* = 5.201703 pF	
*C_S_* = 1.8 pF	*C_S_* = 1.8085649 pF	45
*C_P_* = 7.5 pF	*C_P_* = 7.569376 pF	

**Table 4:** Capacitor values obtained from DNN for ideal inputs.

Ideal Waveform	DNN Output	Sample Number
Square (0.9–1.0 GHz)	*C_S_* = 2.74568 pF *C_P_* = 13.05057 pF	1
Triangular (1.1–1.2 GHz)	*C_S_* = 1.5721719 pF *C_P_* = 13.184719 pF	2
Square (1.3–1.4 GHz)	*C_S_* = 1.0054374 pF *C_P_* = 7.1495605 pF	3

**Table 5:** Comparison of machine learning methods for impedance matching.

Study	Method	Array Geometry	Neural Network Size	Application	Network Type
[20]	Back-propagation neural network	1D	5 (3 hidden layers)	Wireless power transfer	Gamma matching
[21]	Feedforward neural network	1D	12 (10 hidden layers)	Wireless power transfer	Three cascading L-type stages
This study	DNN	1D	12	Antenna	Gamma matching

## Data Availability

Not applicable.
